# A Journey to the Core of the Plant Cell Cycle

**DOI:** 10.3390/ijms23158154

**Published:** 2022-07-24

**Authors:** Crisanto Gutierrez

**Affiliations:** Centro de Biologia Molecular Severo Ochoa, CSIC-UAM, Nicolas Cabrera 1, Cantoblanco, 28049 Madrid, Spain; cgutierrez@cbm.csic.es

**Keywords:** cell cycle, cell division, retinoblastoma, DNA replication, geminivirus, Arabidopsis, plant

## Abstract

Production of new cells as a result of progression through the cell division cycle is a fundamental biological process for the perpetuation of both unicellular and multicellular organisms. In the case of plants, their developmental strategies and their largely sessile nature has imposed a series of evolutionary trends. Studies of the plant cell division cycle began with cytological and physiological approaches in the 1950s and 1960s. The decade of 1990 marked a turn point with the increasing development of novel cellular and molecular protocols combined with advances in genetics and, later, genomics, leading to an exponential growth of the field. In this article, I review the current status of plant cell cycle studies but also discuss early studies and the relevance of a multidisciplinary background as a source of innovative questions and answers. In addition to advances in a deeper understanding of the plant cell cycle machinery, current studies focus on the intimate interaction of cell cycle components with almost every aspect of plant biology.

## 1. Introduction

The iterative mode of plant organogenesis during postembryonic growth requires a continuous supply of new cells. Therefore, the cell division cycle is not only of primary relevance but also a process strictly coordinated with morphogenesis. It is generally accepted that the cell division cycle results from the occurrence of processes, highly regulated in time and space, leading to the production of two daughter cells. In all cases, genome duplication occurs during S-phase and genome (chromosome) segregation during mitosis (M), that includes cytokinesis to separate the two division products. S and M are separated by the pre-replicative G1 and the post-replicative G2 phases. Progression through S and M is dictated by the activity of cyclin-dependent kinases (CDKs)/cyclin complexes, which is regulated mainly at the quantitative level [1]. CDK activity also depends on the nature of cyclins and their availability, the presence of CDK inhibitors, and the activation/inhibition of the CDK moiety by protein phosphorylation and phosphatase balance. 

Advances in gene discovery clearly supported the conclusion that the eukaryotic cell cycle machinery has been highly conserved throughout evolution from the unicellular ancestors to the current variety of multicellular forms of life. The Arabidopsis genome encodes for ~40 cyclins, a variety of CDK inhibitors (KRPs, SIM and SMR) and various CDK activating kinases and phosphatases.

In addition, there are other cyclic events coordinated with those occurring throughout these phases, such as the CDK/cyclin activity peaks, frequently in mid/late G1 and late G2, cytokinesis, centromere maturation, chromatin remodeling, among others. All of them are highly regulated to function in an unidirectional manner [1,2], as a consequence of several redundant or complementary pathways, e.g., gene expression, subcellular localization, post-translational modifications or targeted proteolysis. In the case of plants, the cell cycle is coordinated with morphogenesis by the direct interaction of cell cycle regulators with plant-specific cell fate pathways. However, there are a number of features that are universal to all eukaryotic cell cycles. 

## 2. Where Do We Come From?

Why are we interested in plant cell cycle, DNA replication and chromatin? Understanding the reasons to launch a research project to study various aspects of cell proliferation in plants in 1993 requires a few considerations. It was not the result of a Eureka moment in the middle of a night dream, as advances in science are frequently presented. The main reason to choose a model and a project with a long-term projection was the consequence of a previous background in apparently disparate topics, several in our case. These go deep into several years before launching the project, when I had a direct experience with different research interests and model systems, something that with the perspective of time, I see as a very useful and important source of freedom to tackle scientific questions. 

During my PhD, I was trained in a plant cell biology laboratory, then I worked on DNA repair in plants (see also below) and wanted to gain experience in molecular biology in mammalian cells. In the mid-1980s, I was studying the molecular basis of eukaryotic DNA replication, in particular the initiation steps, using animal oncoviruses, mainly SV40 as a model, in the laboratory of Mel DePamphilis (https://www.nichd.nih.gov/research/atNICHD/Investigators/depamphilis; accessed on 20 July 2022). The SV40 genome is a small double-stranded DNA (dsDNA) circular molecule that replicates in the nucleus of the infected mammalian cells, where it associates with host nuclear histones to form nucleosomes. Therefore, at that time, SV40 as well as other oncoviruses such as adenoviruses and polyomaviruses were considered the most suitable and amenable model systems to study eukaryotic DNA replication. Initiation of SV40 DNA replication requires a single virally encoded protein, the T-antigen (T-ag), that also regulates transcription of other viral genes [3,4]. Using reconstitution experiments, various laboratories established in vitro DNA replication assays, which were crucial for the identification of many cellular components involved in viral DNA replication such as several DNA polymerases and accessory factors, PCNA, RFC, RPA, among others. We focused on the interaction of various forms of T-ag with the SV40 DNA replication origin [5], studies that later were instrumental in the initial stages of our laboratory. The SV40 T-ag was also well-known as a key viral protein that stimulates proliferation of the infected cell, and establishes a host cell environment full of cellular factors suitable for efficiently complete the viral replication cycle.

While still working in this project, J. M. Sogo (now retired from the Institute of Cell Biology in Zürich) spent a sabbatical stay in the DePamphilis laboratory. I joined him in a project using psoralen crosslinking and electron microscopy strategies, which were developed by him, to study how the SV40 DNA molecule associates with histones to form nucleosomes [6]. These studies deeply influenced our interest not only in chromatin dynamics but also in DNA-protein complexes during my time in the laboratory of Margarita Salas, back in Madrid [7,8]. This provided a useful background in protein-DNA interactions and electron microscopy, also very relevant at the initial stages of our laboratory. In fact, acquiring a background in different disciplines is beneficial not only to approach question from complementary perspectives but also to become more versatile in the daily laboratory life. Regarding the relevance and advantage of having a multidisciplinary background, I would like to quote Ardem Patapoutian, awarded the Nobel Prize in Physiology or Medicine in 2021: “Perhaps the most important decision for scientists is to decide which questions to ask. I’ve found it helpful to talk to people outside my immediate field. Explaining the impact of your research to a broader audience is a strong litmus test”.

## 3. Early Studies

The decision to choose a topic to launch a long-term independent project takes time for reflection, and certainly it is permeated and enriched by various inputs. I remember trying to identify a model system where I could apply my previous background and enter a field with sufficient freedom to ask questions from a different, sometimes unusual, perspective. I thought of combining approaches to integrate DNA-protein complexes, DNA replication and cell proliferation, ideally in a viral system that looked relatively simple, where I could establish a suitable scientific niche. It was not trivial, but in the early 1990s, I started to learn about geminiviruses, a family of relatively poorly known plant viruses of small ssDNA genomes that encode only a few proteins and replicate in the host cell nucleus [9] through dsDNA intermediates that could form nucleosomes [10]. My impression was that these plant viruses really looked like SV40! 

I attended a seminar in 1992 delivered by Philip Mullineaux (https://www.essex.ac.uk/people/mulli06704/philip-mullineaux; accessed 20 July 2022), who at that time worked at the John Innes Centre in Norwich, in which he reported studies with the wheat dwarf geminivirus (WDV). It was a pleasure and very enlightening to have a long discussion with him about geminivirus biology. I soon recognized the potential of geminiviruses as excellent models to tackle the kind of questions I had in mind to define mechanisms of initiation of DNA replication and also how the viral proteins functionally interacted with the host machinery. This was a convincing set of arguments to justify launching a project based on geminivirus biology. A short stay in the Mullineaux’s laboratory and his generosity to provide wheat cell cultures, viral vectors and other materials facilitated enormously the initial experiments on two complementary roads: one focused on the mechanisms of viral DNA replication and another on virus–host cell interactions, in particular, with the host cell proliferation machinery. 

Regarding the mechanism of WDV genome replication at the dsDNA stage, we applied our SV40 background to identify minimal sequences required for initiation of DNA replication, their DNA sequence-based structural properties, the protein complexes formed by the early proteins RepA and Rep, and the host cell proteins loaded after the initial step of Rep-mediated nicking [11,12,13,14]. Further studies on various members of the geminivirus family have been reviewed elsewhere [15,16,17]. The early days in our laboratory were also marked by a close relationship with Eduardo Rodriguez-Bejarano (https://www.ihsm.uma-csic.es/investigadores/27; accessed 20 July 2022), who organized the 1st International Symposium on Geminivirus (Almeria, 1994) and was very kind to invite us to participate, present our work and introduce our new lab to the geminivirus world.

The field of DNA replication initiation complexes in eukaryotic viruses and cells was hot at that time, and I recall a key presentation in a CSHL meeting where the Stillman’s laboratory presented the identification of the Origin Recognition Complex (ORC), the putative initiator complex, functionally equivalent to the SV40 T-ag [18]. Everyone thought that our understanding of how eukaryotic cells initiate DNA replication was solved. It was certainly a seminal work, although it has taken several decades to obtain a more detailed account of its complexity and how far we still are from fully understanding how the interaction of ORC and DNA replication origins work, in the context of a replicating chromatin molecule [19,20].

In line with this discovery and our interest in viral and cellular DNA replication, we initiated a long-term project to identify components of the initiation of DNA replication in plant cells. It is worth keeping in mind that this was years before any plant genome sequences were available, and therefore, the effort relied on screening EST (expression sequence tags) collections and cDNA libraries by Southern blots and PCR amplification based on DNA sequence information of putative plant homologs. This led us to isolate cDNA clones encoding several proteins involved in the formation of the pre-replication complexes (pre-RC), such as CDC6, CDT1 and ORC1 [21,22,23] (see also below). 

The other laboratory project was aimed at understanding the molecular basis of the functional interaction of geminiviruses with the host cell cycle machinery. This was virtually a black box, although it was known that replicative forms of geminiviruses were abundant in S-phase cells [24]. This side of the original project had deep roots into (i) my early work of cell proliferation kinetics and DNA repair in *Allium cepa* meristems [25,26,27,28,29], and (ii) my previous experience with SV40 T-ag interactions with the host cell. The identification in 1987 of the human homolog of the yeast p34Cdc2 protein [30], the main cyclin-dependent kinase (CDK) active during the cell cycle triggered the interest in several laboratories to ask whether plant cells would have a similar protein. This led to the original finding of Cdc2 kinase homolog and its putative cyclin partners of the A- and B-types [31,32,33,34,35,36]. 

The accumulated evidence at that time strongly supported the prevailing view that plants, as evolutionary old organisms, might have a cell cycle control similar to that of yeasts. Our studies of the different domains and amino acid motifs of the WDV RepA early protein pointed to a very different direction. I acknowledge my frequent and highly illuminating discussions with Manuel Serrano (https://www.irbbarcelona.org/en/research/manuel-serrano; accessed 20 July 2022; https://altoslabs.com/team/principal-investigators-cambridge/manuel-serrano/; accessed 20 July 2022), who was then a postdoc with David Beach (CSHL), where he identified p16, the first CDK inhibitor identified in human cells [37]. These discussions on how the cell cycle is triggered by oncoviruses were instrumental to identify a short amino acid motif (LxCxE) in the WDV RepA protein. Remarkably, this motif was also present in the early proteins of animal oncoviruses, responsible for their interaction with the human tumor suppressor retinoblastoma (RB) protein and other so-called “pocket” proteins (p107 and p130). That was highly suggestive that the LxCxE motif of the geminivirus RepA might well be functionally equivalent to that of SV40 Tag and that it might mediate interaction with a putative plant retinoblastoma homolog, a totally unexpected view. It was demonstrated that point mutations in the LxCxE motif of WDV RepA reduced the efficiency of viral DNA replication in cultured wheat cells and, strikingly, expression of human Rb in plant cells was also detrimental for WDV DNA replication [38]. 

Two other publications in 1995 produced an excitement in our laboratory difficult to express with words. One was the identification of D-type cyclins, which share with their animal counterparts an LxCxE motif [39,40]. Another was that geminiviruses triggered an unscheduled S-phase in infected cells [41]. These findings led us to hypothesize that plant cell cycle regulation, in particular the G1 progression, might have evolved early in eukaryotic evolution from an ancient RB-related protein, then maintained and expanded in animal lineages [42,43]. With this conceptual framework and through a collaboration with Greg Hannon (https://www.hannonlab.org/; accessed 20 July 2022), who had access to one of the few maize cDNA libraries, we sought to identify a cDNA clone encoding a plant retinoblastoma-related (RBR), a homolog of human Rb protein [44]. This also paved the way to the identification of the first plant E2F transcription factors [45,46]. I am indebted to Denes Duddits (BRC, Szeged), who invited me to attend an EMBO Workshop that he organized in Szeged on the “Control of cell division in higher plants”, which was key to introducing us to the incipient plant cell cycle community. RBR proteins were later found in other plant species, including unicellular algae, and shown to be essential [42,43,47,48,49,50]. Likewise, E2F family members were characterized [51,52], including the atypical members E2F/DEL, later identified in mammalian cells [53,54,55], as well as their genome-wide targets [56,57]. 

## 4. Where Are We Now?

**Figure 1:**
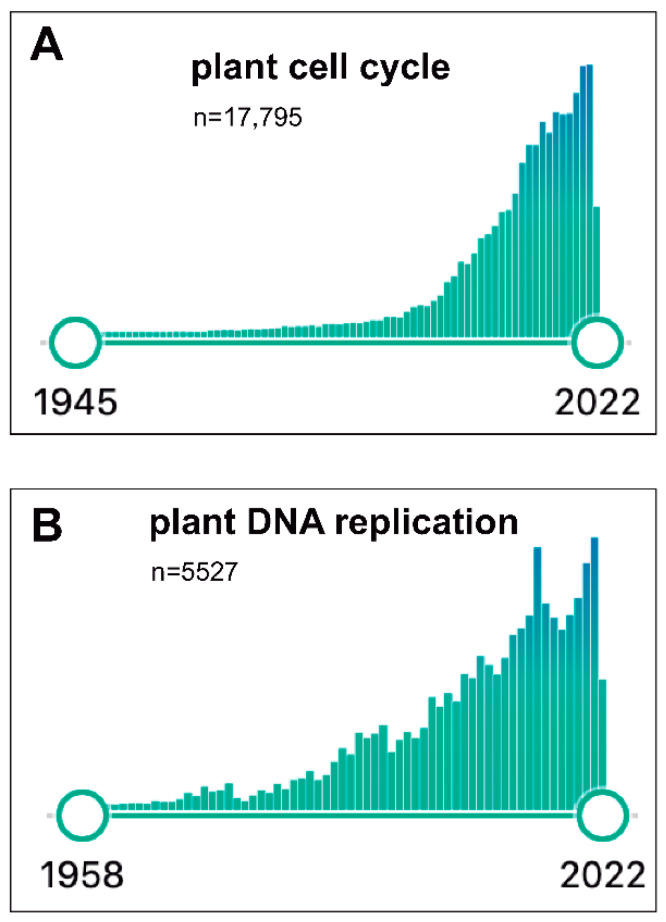
Timeline results of publications recorded in PubMed (June 2022) for the terms “plant cell cycle” (**A**) and “plant DNA replication” (**B**).

Since the mid-1990s, the plant community had been expectant with the possibility of having access to the full genome sequence of *Arabidopsis thaliana*, which finally came to be true [58]. This led our laboratory to leave the geminivirus projects and move fully to Arabidopsis, focusing on several cell cycle aspects. In fact, during the last two decades, an impressive advance has occurred in our understanding of the basic components of the cell cycle machinery, not only in Arabidopsis but also in other plant species. Figure 1A illustrates the exponential interest in plant cell cycle research moving from ~75 publications in 1993 up to >1370 during 2021, with a total of >17,750 publications since 1945. Likewise, the plant DNA replication field has also followed a similar growing pattern, steadily increasing from ~100 publications in 1995 up to ~330 in 2021 (with a total of >5500; Figure 1B). Several laboratories, including ours (where the participation of many outstanding PhD candidates, postdocs and visitors has been crucial), have joined efforts to identify and define the role of cell cycle factors that participate in various stages and how they work coordinately to regulate cell division. I will not present here a detailed account of our current knowledge of mechanistic aspects of plant cell cycle. The interested reader is directed to several comprehensive reviews published recently [43,59,60,61,62], although I would also recommend to have a look at the earlier reviews where the evolution of this field can be easily grasped [63,64,65,66,67,68,69,70]. 

Advances in scientific research are intimately associated not only with new conceptual contributions but also with novel technological approaches and tools. In our case, they have come from two major lines. One consists of tools to assess cell cycle progression by live-imaging, which has been mastered in our laboratory by Bénédicte Desvoyes. By collecting results of different projects dealing with the dynamics of DNA replication proteins and histones during S-phase, a multiple expression cassette was designed containing three markers labeled with different fluorescent tags (CDT1a-CFP, H3.1-mCherry and CYCB1;1-YFP) that allows the identification of all cell cycle phases in different plant organs [71,72]. The PlaCCI marker line was originally developed for Arabidopsis (PlaCCI), but a similar strategy is being transferred to other plant species by different laboratories. The use of PlaCCI in several developmental contexts is confirming its usefulness for cell proliferation studies [73,74,75].

Another came from advances in chromatin biology with the development of highly specific and sensitive tools to identify various chromatin features both genome wide and at specific genomic locations, and related to initiation of DNA replication and transcriptional activity [76,77]. The combination of these tools together with other cell and molecular biology, genetic and genomic strategies constitutes the basis on which our current research projects are supported to tackle several complementary objectives.

### 4.1. Cell Cycle Control during Organ Development

The continuous production of new organs in plant during its postembryonic growth (vegetative and reproductive) requires a sustained supply of new cells that will eventually give rise to all different cell types of a particular organ. These cells are exclusively produced in the meristems, unique plant locations where all the cell proliferation occurs. We have focused on the root apical meristem (RAM), which is located at the root apex, where three major compartments can be distinguished. One is the quiescent center (QC), a group of few cells that divide rarely or in response to organ damage after root stress, and that constitutes an organizing center for the stem cell niche. Surrounding the QC cells are the stem cells that divide asymmetrically to produce one daughter cell that remains in contact with the QC cells and is perpetuated as a stem cell, and another daughter that has a limited proliferation potential. Division of these stem cell daughters during several cell division cycles produces a cohort of cells that constitutes a transit-amplifying compartment, the RAM proper [78].

Cell proliferation kinetics in the RAM has been assumed to be homogeneous with cells dividing at a fast rate, compared with the slow dividing rate of the stem cells. All different cell types located along the RAM have been also considered to develop cell cycles of similar length, following the same exponential kinetics (see discussion in [79]), a view that nonetheless had been challenged long time ago [80]. It is also known that the stem cell cycle is slow [81] and that cells located in the more proximal half of the RAM (in contact with the RAM boundary) possess a smaller probability to divide. This together with direct experimental data [82] gave support to distinguish two domains within the RAM: the proliferation domain (PD), located in the more distal half, and the transition domain (TD), in contact with the RAM boundary [79,83].

In an effort to define cell proliferation kinetics during organogenesis, it was asked whether chromatin dynamics could provide new insights from a different perspective [84]. Thus, by using live-imaging of roots, the incorporation of the canonical histone H3.1-GFP during S-phase and its replacement by the histone variant H3.3-mRFP was followed [85]. A subpopulation of RAM cells, which corresponded to cells undergoing their last cell cycle within the RAM, was identified by its reduced H3.1 level. A massive replacement of H3.1 occurs during the last G2 phase before entering the endocycle program. It was also found that this G2 phase has a ~30% longer duration than the G2 phase of earlier stem cell derivatives, particularly in the two epidermal cell types. There are various possibilities currently being tested to define the mechanism responsible for the G2 lengthening during the last cell cycle in the RAM. It is worth noting that a similar phenomenon has been reported for the last cell cycle in Drosophila embryos [86].

One possible control stage is the accumulation dynamics of CYCB1s and CDKBs, active during G2 that can be modified depending on the position of a cell within the RAM. The analysis of the appearance of EdU-labeled mitosis after an EdU pulse, a read-out of G2 progression, should shed light onto this question. Additionally, a control mechanism could depend on the DREAM complex, originally identified in Drosophila but widespread in plants [87], where it plays different roles in connection with RBR [43,88]. A recent report showed that specific CDK inhibitors act downstream of DREAM in G2 to control cell size [89]. Thus, a reasonable hypothesis is that DREAM complexes might participate in the regulatory network controlling G2 duration along the proximal–distal axis of the RAM.

A detailed analysis of live-imaging recordings has revealed significant differences in cell cycle duration along the RAM axis, mainly dictated by differences in the G1 duration. The long G1 phase is not restricted exclusively to stem cells. Rather, a gradient of G1 durations is established ranging from >20 h of stem cells and their derivatives up to ~1/3 of the RAM to only 1–2 h in cells close to the RAM boundary [90]. This G1 duration gradient conforms to an incoherent feed-forward loop (IFFL) where a driver possesses two opposite functions: one confers cell proliferation potential and another activates the expression of a negative regulator of G1 progression, thus affecting the cell cycle progression rate. One of the components of the “driver” seems to be the PLETHORA (PLT) proteins, since the G1 gradient is abolished in the *plt1*, *plt2* double mutant.

There are several questions that remain unanswered. One is whether there are other genetic pathways that coordinate upstream regulation of PLT function [91] with G1 progression control. Analysis of transcription factors expressed in the RAM revealed a subset that has an expression pattern similar to that of PLT proteins. Another key component of the pathway is the RBR1 protein, since it is known that the unphosphorylated (or underphosphorylated) forms of RBR1 restrict G1 progression. Consistent with this, *rbr1* mutants develop very rapid G1 phases along the entire RAM without any signs of a G1 duration gradient [90]. Therefore, an important question to answer in the future will be to identify how the phosphorylation state of RBR1 affects its repressive function and the phosphorylation sites responsible for restricting G1 progression. Successful development of these studies will constitute a major advance in our understanding of the developmental zonation of the root apical meristem. Differences in cell cycle duration in the proximal–distal axis of the RAM occur in all cell types, that is, across the radial axis. However, we do not know yet how a given cell at a given location in the RAM senses the proliferation status in coordination with its neighbors to produce a G1 duration gradient, something that is being currently explored in our laboratory.

### 4.2. Chromatin Dynamics and Genome Replication during S-Phase

Deposition and/or maintenance of chromatin features, that is, cytosine methylation, histone variants and their post-translational modifications, is strictly regulated throughout the cell cycle. Some of them are related to the transcriptional waves characteristic of G1 and G2, responsible for the synthesis of products required for S-phase and mitosis, respectively. However, others play a function in other cellular processes. The chromatin landscape associated with initiation of DNA replication during S-phase and how they regulate eu- and heterochromatin organization has been a long-standing interest of our laboratory.

All eukaryotic cells initiate replication of their genomes at multiple locations known as DNA replication origins (ORIs). Their location is dictated by the assembly of the pre-replication complexes (pre-RC), a process that occurs early in G1 [19]. Pre-RCs consist of the heterohexameric origin recognition complex (ORC), CDC6, CDT1 and the heterohexameric minichromosome maintenance (MCM) complex. Pre-RC homologs have been now identified in several plant species [21,22,23,92,93,94,95]. Pre-RC assembly is known as licensing of chromatin for replication, but only a subset of licensed chromatin locations marked with assembled pre-RCs are later activated at the G1/S transition by disassembly of the pre-RC and further addition of other replication proteins, thus defining the location of active ORIs. Efforts in different laboratories have identified the genomic location of ORIs in several eukaryotic cell types, including plant cells [76,77,96,97,98,99,100,101,102].

A long-standing question is whether and how the chromatin landscape at and around active ORIs serves to define ORI location and/or their potential to be activated [103]. A detailed map of nine chromatin states (CS) of Arabidopsis has been reported based on the combination of 16 different chromatin features, including DNA base composition, histone types and their post-translational modifications [99]. These chromatin states are highly correlated with the major genomic features: CS1, CS2 and CS3, enriched in H3K4me3 and histone variants H3.3 and H2A.Z, colocalize with TSS, proximal promoters and 5′ end of genes, respectively; CS4 and CS5, both enriched in H3K27me3, mainly correspond to long intergenic regions and Polycomb-regulated genes; CS6 and CS7 are associated with 3′ end of genes and long genes, respectively; finally, CS8 and CS9, enriched in C methylation, H3K9me2 and H3K27me1, correspond to the two types of heterochromatin, AT-rich and GC-rich, respectively [104].

Detailed analysis of ORI location in developing Arabidopsis seedlings in relation to these chromatin states revealed that they can occur in any CS [77]. However, a high preference for CS1-2-3 is detected, whereas ORIs tend to be less represented in CS4, CS5, CS8 and CS9. These results suggested that ORIs have a preference for regions of open and accessible chromatin, as it occurs in other eukaryotes [105]. Studies using a different approach have mapped ORIs in AT-rich genomic locations [106]. A future challenge is to identify the molecular determinants defining ORI activity. Since ORIs have been detected in genomic regions with very different chromatin landscape, it is conceivable that a single chromatin mark, as originally hypothesized, is not sufficient to confer ORI activity, although it might be necessary. It is also conceivable that ORI located in closed chromatin regions, e.g., heterochromatin, might prefer some regions with a relatively less compact chromatin feature. This has been tested by analyzing ORIs located in Arabidopsis transposon elements (TEs). It has been found that active ORIs associated with TEs tend to be located in GC-regions, where Gypsy family TEs are located and, among them, tend to have a higher GC content [107].

It is well established that embryonic cells develop very rapid S-phases because they possess a larger set of functional ORIs than differentiated cells [108]. One attractive aspect to explore in the future is whether modifying the active ORI set has an impact on cell growth performance and cell proliferation potential. Could changes in the ORI location pattern have an impact at the cellular and developmental level? In the case of plants, this could be particularly relevant given their regeneration capacity and the challenges faced by the changing environmental conditions, an aspect that is actively studied in our laboratory.

A particularly case interesting is that of the ORC1 proteins encoded by two genes, *ORC1a* and *ORC1b*, in Arabidopsis. By studying the phenotypes of single *orc1a-2* and *orc1b-2* mutants, we have found that they play distinct functions. The knockout *orc1b-2* mutant is viable under normal growth conditions but is severely impaired after a treatment with the DNA polymerase inhibitor aphidicolin, likely due to its deficiency in the amount of pre-RC complexes assembled. On the contrary, the *orc1a-2* mutant shows a wild-type phenotype with and without aphidicolin and apparently normal heterochromatic chromocenters. However, they show defects in deposition of the typical heterochromatic mark H3K27me1, while maintaining normal levels of H3K9me2 [109]. H3K27me1 is deposited by the histone methyltransferases ATXR5 and ATXR6 [110,111], cell cycle-regulated enzymes with a peak of expression in S-phase [112]. Establishment and maintenance of heterochromatin is crucial for genome integrity during development [113,114] and ATXR5 and ATXR6 function redundantly to suppress over-replication of heterochromatin [110]. It has been reported that mutations in these two genes cause a reduction of ~20% in H3K27me1 levels, in addition to heterochromatin decondensation and upregulation of TE expression [110,115]. Therefore, it is likely that ORC1a, but not ORC1b, somehow facilitates ATXR5/6 activity, although the precise mechanism is yet to be defined.

### 4.3. Perspectives on Other Cell Cycle Topics

The general strategies of plant cell cycle and the cellular machinery are much more similar to the animal cell cycle than to yeast. However, one of the more striking features of the plant cell cycle is the very large amount of core cell cycle components. Thus, in Arabidopsis 8–9 CDKs, >40 cyclins, >20 CDK inhibitors or 4 RBR proteins in many monocots, to cite some examples, have been identified (https://www.araport.org/; https://www.arabidopsis.org/; accessed 20 July 2022). Initial genome-wide transcriptomic studies of cell cycle genes indicated that many of them were expressed in Arabidopsis cultured cells [116], which was initially considered as a case of functional redundancy. Later studies revealed, however, that different sets or components of the cell cycle machinery had a cell type-, growth- and/or developmental stage-specific expression pattern. As a consequence, cell cycle regulators have been identified now as targets of a plethora of signaling pathways controlling many aspects of plant physiology [62,117].

Plant growth under normal and stress conditions is the result of a complex network of signaling pathways. Abiotic stress is associated with specific changes in cell proliferation and gene expression as well as heterochromatin disorganization and transposon reactivation, all having a significant impact on plant genomes and growth [118,119]. A current focus is on histone dynamics in both euchromatin and heterochromatin maintenance in relation to environmental factors [120]. An attractive hypothesis is that, in the long term, deposition of various histone forms and the activity of chromatin modifying enzymes at specific genomic locations could be targeted to improve plant tolerance to abiotic stress, genome stability and cell division potential. 

Organogenesis requires not only the production of new cells of a given type but also the occurrence of formative cell divisions to produce daughter cells that eventually will take different cell fates and differentiation pathways. Thus, cell fate decisions are needed in many plant locations during the entire life of the plant, including embryogenesis [74]. Control of formative divisions, frequently through asymmetric cell division (ACD), directly involves specific cell cycle regulatory factors. Thus, Aurora (AUR) kinases possess RBR1 binding motifs, highlighting a novel possible role of RBR1 in formative division and the machinery involved in cell plate orientation are examples of the importance during formative cell divisions [121,122,123].

Once the newborn cells are produced, they must decide whether they maintain the same fate as their mothers or acquire a new one. They possess a window during the cell cycle to acquire a new fate, a process that is not fully understood regarding how this decision occurs during the cell cycle. The end of mitosis and early stages of G1 seems appropriate, since changes in chromatin accessibility have been reported, for example, in the trichoblast/atrichoblast decision in the root epidermis [124]. Therefore, much research is needed to identify the cell cycle regulatory factors and parameters that affect cell fate decisions at different developmental stages. In the context of a growing organ, it is of primary relevance to understand the control of cell division potential [125,126] and the positional dependence of cell cycle phase duration [85,87,90,91,127].

The average cell size varies among different cell types, normally associated with the cell fate decisions taken, yet they follow similar rules for cell division. Cell size control during the cell cycle is intimately linked to cell cycle progression, whereby specific cell cycle components are involved, e.g., KRP4 used as a cell size monitoring molecule [73]. A topic of special relevance is cell size control during the endocycle [128,129], when cells duplicate their genome without an intervening mitosis, since the presence of endoreplicated cells is frequent in many plant species.

The high capacity to regenerate organs is one of the most striking features of many plants. This depends on the presence of long-lived stem cells, the capacity to reprogram somatic cells into pluripotent cells and the action of hormonal signals [126]. The response to hormonal signals is also directly linked to the activity of specific cell cycle regulatory factors, e.g., RBR1 in the auxin/cytokinin balance in the root [130,131], the ethylene response through ERF115 [132,133], the brassinosteroid signaling through BRAVO and CCS52A2 [134,135,136]. The interplay of hormonal signals with nutrient availability and metabolism is another field that should benefit from being analyzed from a cell proliferation perspective [137,138].

As an insider witness of plant cell cycle research over the past four decades, I can confidently confirm that the combination of cellular, molecular, developmental, genetic and genomic approaches has led to a brilliant growth of this field. Now the cell cycle components are no longer considered only part of a machine to produce two daughter cells but as true hubs that network with a large variety of processes crucial for the life of plants. As the scope of plant cell cycle research expands, we can only see new exciting discoveries. These should range from detailed mechanistic aspects of how cell cycle components act to cytoplasmic and nuclear signaling cascades, translational control, chromatin dynamics involved in replication and transcription, novel interactions with plant developmental processes and interaction with the environment.

## Data Availability

Not applicable.
